# Multimodal imaging findings of sarcomatoid carcinoma of the urinary bladder

**DOI:** 10.1002/ccr3.8761

**Published:** 2024-04-09

**Authors:** Guiwu Chen, Xiaoling Leng, Su Yu, Wenqin Liu, Xiaomin Liao

**Affiliations:** ^1^ Department of Ultrasound The Tenth Affiliated Hospital of Southern Medical University, Dongguan People's Hospital Dongguan China; ^2^ Department of Pathology The Tenth Affiliated Hospital of Southern Medical University, Dongguan People's Hospital Dongguan China

**Keywords:** magnetic resonance imaging, positron emission tomography‐computed tomography, sarcomatoid carcinoma, ultrasonography, urinary bladder neoplasms

## Abstract

Sarcomatoid carcinoma, a rare and aggressive subtype of bladder cancer, accounting for 0.3% of cases, is more aggressive than urothelial carcinomas. Accurate diagnosis, crucial for treatment, can be challenging. We present a characterized case of sarcomatoid carcinoma of the urinary bladder using multimodal imaging and pathology.

## Graphical abstract

A 64‐year‐old man diagnosed with sarcomatoid carcinoma of the urinary bladder underwent comprehensive evaluation utilizing multiple imaging modalities, such as ultrasound, magnetic resonance imaging, and positron emission tomography‐computed tomography, which were subsequently validated through pathological examination.

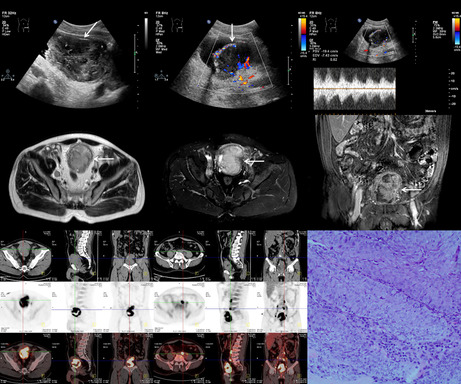

Sarcomatoid carcinoma is a rare and aggressive subtype of primary bladder cancer that accounts for approximately 0.3% of all primary bladder tumors. The clinical presentation of sarcomatoid carcinoma of the urinary bladder is similar to that of urothelial carcinomas.^1^ However, sarcomatoid carcinoma is exceedingly uncommon and more aggressive than urothelial carcinomas. Accurate diagnosis of this rare variant is essential but can be challenging.^2^ Here, we present a case of sarcomatoid carcinoma of the urinary bladder that was characterized using multimodal imaging and confirmed by pathological examination.

A 64‐year‐old man presented to our hospital complaining of progressive difficulty in urinating, accompanied by hematuria for 3 years. Upon laboratory examination, complete blood count and specific tumor markers were normal (red blood cell: 3.36 × 10^12^/L, white blood cell: 15.1 × 10^9^/L, platelet count: 392 × 10^9^/L, alpha‐fetoprotein: 2.47 ng/mL, carcinoembryonic antigen: 2.89 ng/mL, carbohydrate antigen: 19–9 10.39 U/mL, and prostate‐specific antigen: 1.35 ng/mL). Abdominal ultrasound revealed a solid mass protruding into the bladder cavity, with inhomogeneous internal echoes and hyperechoic periphery (Figure 1A). The mass exhibited abundant blood flow signals (Figure 1B) with a resistance index of 0.62 (Figure 1C). Magnetic resonance imaging detected the mass was papillary and ill‐defined with slightly longer signals on both T1‐weighted (Figure 2A) and T2‐weighted imaging (Figure 2B) as well as a significant enhancement (Figure 2C). Positron emission tomography‐computed tomography demonstrated that the irregular mass exhibited heterogeneous density and striped calcification, with a CT value of 37 HU (Figure 3A). Additionally, multiple enlarged lymph nodes were observed in the lower abdomen, adjacent to the abdominal aorta and bilateral iliac vessels. These lymph nodes exhibited abnormal radioactive distribution, with a maximum standard uptake value of 22.1 (Figure 3B). Finally, the patient underwent a cystoscopic biopsy, and the pathological diagnosis of sarcomatoid carcinoma was confirmed. Hematoxylin and eosin staining exhibited heterotypic cell proliferation with patchy, infiltrative growth patterns. Large, deeply stained nuclei and visible nucleoli were present, along with notable heterotypicity and necrotic tissue (Figure 4). Immunohistochemical staining results were positive for Vim, GATA, Bob.1, and negative for CK, EMA, Pax‐5, CD30, ALK(1A4), Desmin, SMA, HMB‐45, Melan A, and S‐100. The Ki‐67 index was approximately 70%. The patient made a decision to decline the surgical procedure and was subsequently released from the hospital. Half a month later, the patient had to be readmitted as a result of urinary retention. Unfortunately, following the patient's discharge again, we were unable to establish contact for further follow‐up.

**FIGURE 1:**
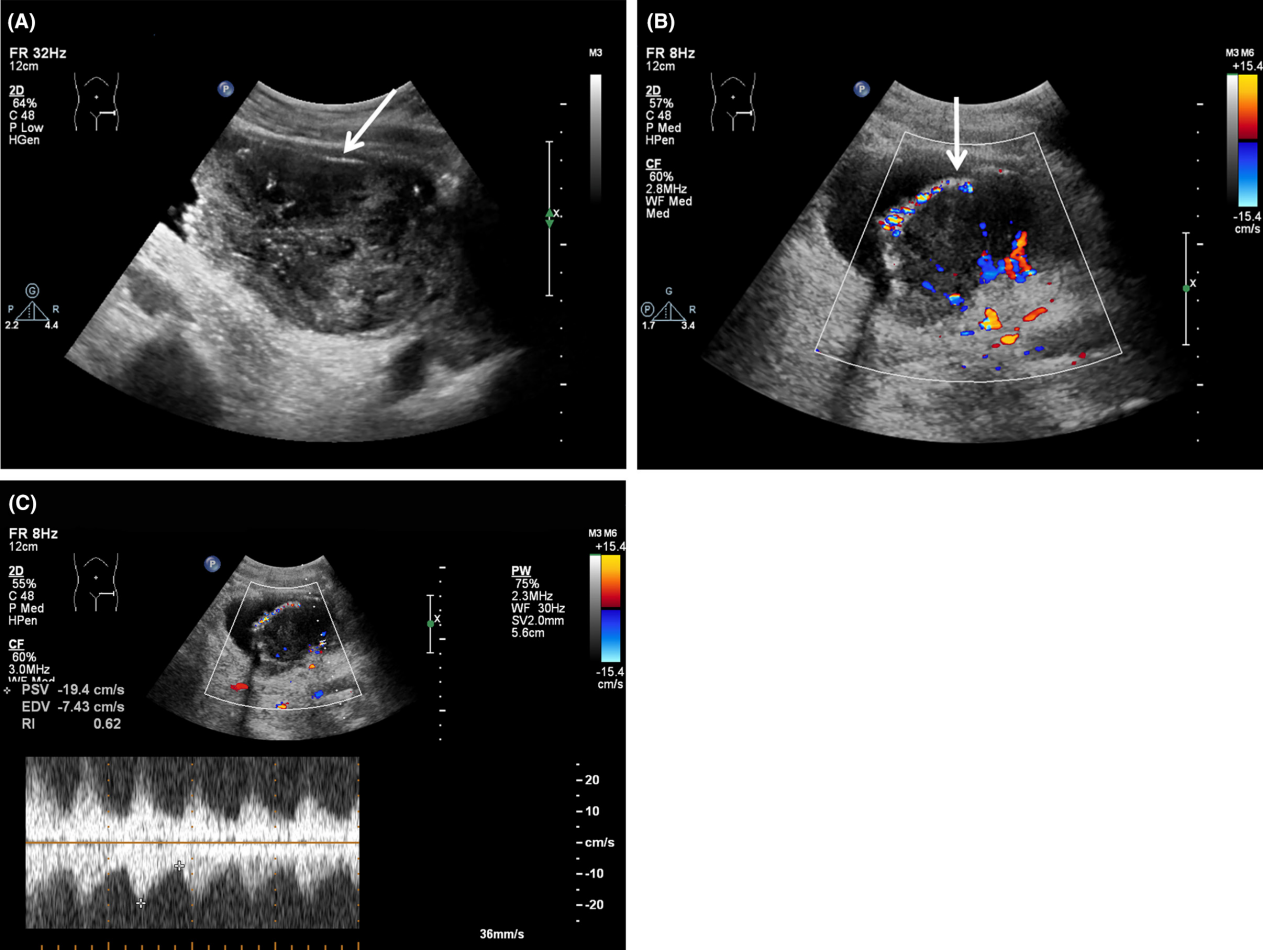
Abdominal ultrasound of sarcomatoid carcinoma of the urinary bladder. (A) Grayscale ultrasound showed a solid mass (arrow) with inhomogeneous internal echoes and hyperechoic periphery. (B) Color Doppler flow imaging showed abundant blood flow signals within the mass (arrow). (C) Pulse wave Doppler ultrasound showed a resistance index of 0.62.

**FIGURE 2:**
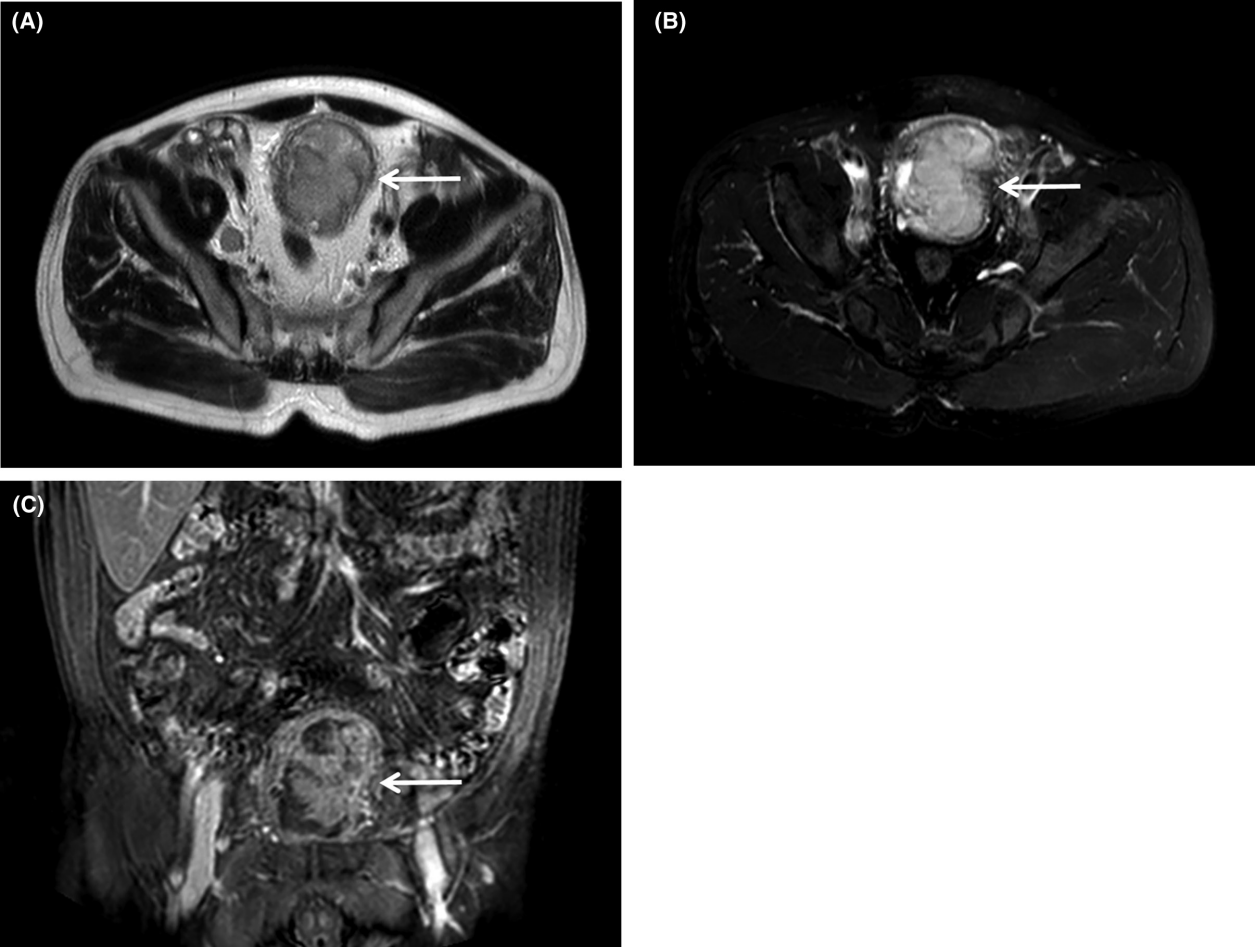
Magnetic resonance imaging of sarcomatoid carcinoma of the urinary bladder. (A) T1‐weighted imaging showed the mass (arrow) with slightly longer signals in the axial section. (B) T2‐weighted imaging showed the mass (arrow) with slightly longer signals in the axial section. (C) The enhanced scanning showed the mass (arrow) with a significant enhancement in the coronal section.

**FIGURE 3:**
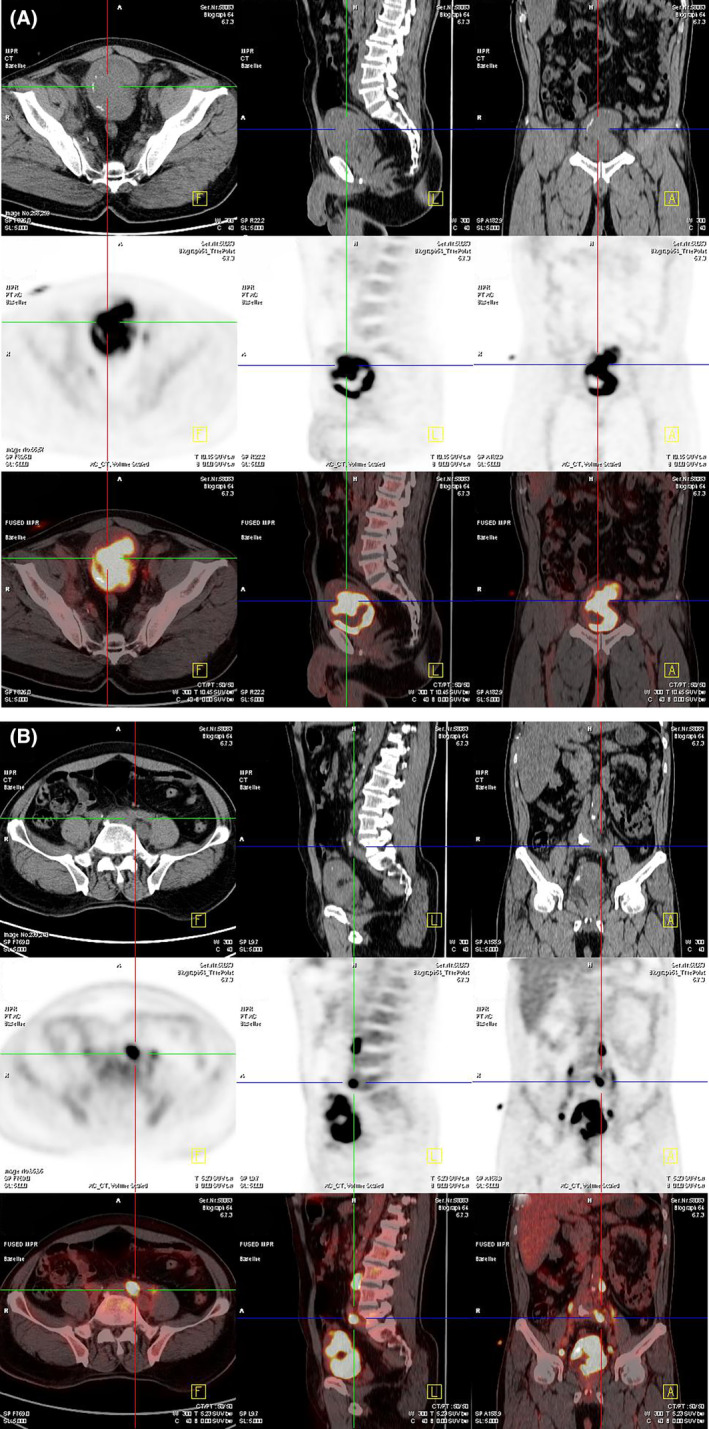
Positron emission tomography‐computed tomography of sarcomatoid carcinoma of the urinary bladder. (A) Positron emission tomography‐computed tomography demonstrated that the irregular mass exhibited heterogeneous density and striped calcification. (B) Multiple enlarged lymph nodes were observed in the lower abdomen, adjacent to the abdominal aorta and bilateral iliac vessels.

**FIGURE 4:**
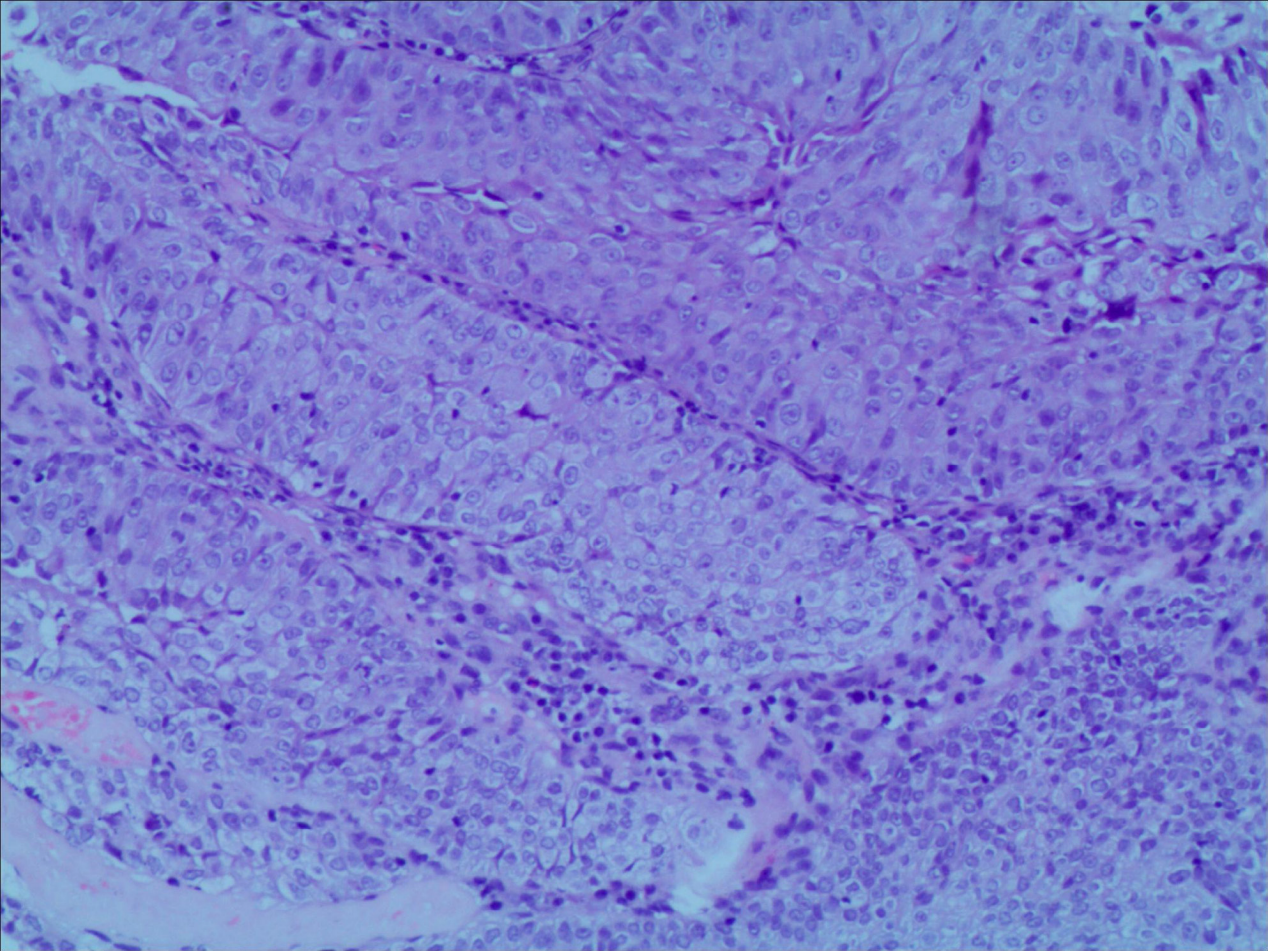
Pathology examination of sarcomatoid carcinoma of the urinary bladder. Hematoxylin and eosin staining exhibited heterotypic cell proliferation with patchy, infiltrative growth patterns (HE ×40).

Sarcomatoid carcinoma is a malignant tumor that exhibits a dual differentiation of both epithelial and mesenchymal tissues. It typically manifests at a mean age of 66 years and is more prevalent in males.^3^ Typical symptoms include gross hematuria, dysuria, pollakiuria, and urinary tract infections.^4^ In our case, sarcomatoid carcinoma was thoroughly characterized using various imaging techniques, including ultrasound, magnetic resonance imaging, and positron emission tomography‐computed tomography, which provided valuable reference information. It is crucial to distinguish sarcomatoid carcinoma from a range of other tumor types, such as papillary tumors, leiomyomas, inflammatory myofibroblastic tumors, urothelial carcinoma, and squamous cell carcinoma, among others. Among these, urothelial carcinoma deserves special attention as it can exhibit similarities in characteristics that may complicate the diagnostic process. Nevertheless, the imaging evidence remains limited, necessitating further research.

## AUTHOR CONTRIBUTIONS

**Guiwu Chen:** Writing – original draft. **Xiaoling Leng:** Supervision; writing – review and editing. **Su Yu:** Data curation; investigation. **Wenqin Liu:** Data curation; investigation. **Xiaomin Liao:** Data curation; investigation.

## FUNDING INFORMATION

No funding was received for this study.

## CONFLICT OF INTEREST STATEMENT

The authors declare no conflicts of interest.

## CONSENT

Written informed consent was obtained from the patient to publish this report in accordance with the journal's patient consent policy.

## TRANSPARENCY STATEMENT

We can confirm that this manuscript is an honest, accurate, and transparent account of the case being reported and that no important aspects of the case have been omitted.

## Data Availability

The data used to support the findings of this study are available from the corresponding author upon request.
